# Impact of vascular endothelial growth factor gene-gene and gene-smoking interaction and haplotype combination on bladder cancer risk in Chinese population

**DOI:** 10.18632/oncotarget.15287

**Published:** 2017-02-11

**Authors:** Dian Fu, Ping Li, Wen Cheng, Feng Tian, Xiaofeng Xu, Xiaoming Yi, Chaopeng Tang, Yongzhong Wang, Quansheng Hu, Zhengyu Zhang

**Affiliations:** ^1^ Department of Urology, Nanjing Jinling Hospital, Nanjing University School of Medicine, Nanjing, Jiangsu, China; ^2^ Department of Urology, The Second Affiliated Hospital of Dalian Medical University, Dalian, Liaoning, China; ^3^ Department of Urology, The First People's Hospital of Huoqiu City, Huoqiu, Anhui, China; ^4^ Department of Urology, Southwest Hospital of the Third Military Medical University, Chongqing, China

**Keywords:** bladder cancer, vascular endothelial growth factor, single nucleotide polymorphisms, interaction, smoking

## Abstract

**Aims:**

To investigate the association of single nucleotide polymorphisms (SNPs) within vascular endothelial growth factor (VEGF) gene polymorphisms, additional gene- gene and gene- smoking interactions with bladder cancer risk.

**Results:**

Bladder cancer risk was significantly higher in carriers of the rs699947- A allele within VEGF gene than those with rs699947- CC genotype (CA+ AA versus CC), adjusted OR (95%CI) = 1.70 (1.16–2.31), and higher in carriers of the rs833052- A allele of within VEGF gene than those with rs833052- CC genotype (CA+ AA versus CC), adjusted OR (95%CI) = 1.65 (1.23–2.12). GMDR analysis indicated a potential interaction between rs2010963 and smoking on bladder cancer risk. Current smokers with rs2010963- GC+CC genotype within VEGF gene have the highest bladder cancer risk, compared to never smokers with rs2010963- GG genotype within VEGF gene, OR (95%CI) = 3.25 (1.71–4.83). Haplotype containing the rs2010963- C and rs833052- A alleles were associated with a statistically increased bladder cancer risk, OR (95%CI) = 2.21 (1.12–3.42).

**Materials and Methods:**

Generalized multifactor dimensionality reduction (GMDR) was used to screen the best interaction combination among SNPs and smoking. Logistic regression was performed to investigate association of 6 SNPs within VEGF gene, additional gene- gene and gene- smoking interaction with bladder cancer risk.

**Conclusions:**

We found that the A allele of rs699947 and the A allele of rs833052 within VEGF gene, interaction between rs2010963 and smoking, haplotype containing the rs2010963- C and rs833052- A alleles were all associated with increased bladder cancer risk.

## INTRODUCTION

As we all known that bladder cancer was an important health problem, and was the 7th and 17th most frequent cancer in males and females, respectively [1], and in China, the occurrence of bladder cancer has dramatically increased from 1991 to 2009, and it accounted for more than 20,000 deaths in China in 2009 [2, 3]. Some risk factors of bladder cancer have been established, such as tobacco smoking and occupational exposure to chemical carcinogenesis [4]. However, only a few of the exposed individuals develop bladder cancer in their lifetime, suggested that genetic factor may also play a crucial role in the pathogenesis of bladder cancer [5].

Vascular endothelial growth factor (VEGF) is a potent endothelial cell-specific regulator of angiogenesis, and has been identified as a key molecule in promoting angiogenesis involving tumor growth and metastasis [6]. The VEGF gene is located on chromosome 6p21.3 and consists of 8 exons. To date, many single-nucleotide polymorphisms (SNPs) of VEGF have been reported [7, 8], and some SNPs have been reported association with susceptibility to several types of tumors, including renal and gastric cancers [9, 10]. Recently, some studies focused on the association between VEGF gene polymorphisms and bladder cancer risks were conducted, but these studies concluded inconsistent results. Several large cohort studies in Europe and the USA have demonstrated that cigarette smoking was an important risk factor for bladder cancer [11, 12], and several genes have been reported interaction with smoking on bladder cancer susceptibility, such as XPC-PAT gene [13], however, no study focused on impact of interaction between VEGF gene and smoking on bladder cancer risk. So the present study aimed to evaluate the influence of VEGF SNPs, possible gene- gene, gene- smoking interaction and haplotype combinations on bladder cancer risk base on a Chinese population.

## RESULTS

A total of 1088 participants (733 males, 355 females) were selected, including 360 bladder cancer patients and 728 control participants. The mean age of all participants was 62.1 ± 13.2 years. General characteristics of 1088 study participants in case and control group were shown in Table 1. The means of age and BMI, and distributions of males and alcohol drinkers were not significantly different between cases and controls. The rate of smokers was higher in cases than that in controls.

**Table 1:** General characteristics of 1088 study participants in case and control group

Variables	Case group (*n* = 360)	Normal group (*n* = 728)	*p*-values
Age (year) (Means ± SD)	62.3 ± 14.1	61.8 ± 14.7	0.593
Males, N (%)	248 (68.9)	485 (66.6)	0.453
Smokers, N (%)	201 (55.8)	257 (35.3)	< 0.001
Alcohol drinkers, N (%)	139 (38.6)	246 (33.8)	0.118
BMI (kg/m^2^) (Means ± SD)	23.6 ± 9.8	24.1 ± 9.2	0.409
Family history of tumor N (%)	56 (15.6)		
Tumor size (cm)			
< 1	84 (23.3)	N/A	
1–3	174 (48.3)	N/A	
> 3	102 (28.3)	N/A	
Stage			
Ta	78 (21.7)	N/A	
T1	181 (50.3)	N/A	
T2	101 (28.1)	N/A	
Grade			
G1	121 (33.6)	N/A	
G2	74 (20.6)	N/A	
G3	165 (45.8)	N/A	

All genotypes are distributed according to Hardy–Weinberg equilibrium in controls. The frequencies for the A allele of rs699947 and the A allele of rs833052 within VEGF gene were significantly higher in cases than that in control group (31.3% *vs*20.4%, 30.0% *vs*19.5%). Logistic regression analysis showed that bladder cancer risk was significantly higher in carriers of the rs699947-A allele within VEGF gene than those with rs699947-CC genotype (CA+ AA versus CC), adjusted OR (95%CI) =1.70 (1.16–2.31), and higher in carriers of the rs833052- A allele within VEGF gene than those with rs833052- CC genotype (CA+ AA versus CC), adjusted OR (95%CI) = 1.65 (1.23–2.12). However, we did not find any significant association of the others SNPs within VEGF gene with bladder cancer risk after covariates adjustment. (Table 2)

**Table 2:** Genotype and allele frequencies of 6 SNPs between case and control group

SNP	Genotypes and Alleles	Frequencies N (%)		OR (95%CI)*	*P*- values	HWE test for controls
		Control (*n* = 728)	Case (*n* = 360)			
rs2010963 (+405 G>C)						
	Co-dominant					
	GG	425 (58.4)	188 (52.2)	1.00 (ref)		0.941
	GC	262 (36.0)	141 (39.2)	1.24 (0.82–1.88)	0.325	
	CC	41 (5.6)	31 (8.6)	1.52 (0.78–2.31)	0.321	
	Dominant					
	GG	425 (58.4)	188 (52.2)	1.00 (ref)		
	GC+CC	303 (41.6)	172 (47.8)	1.34 (0.80–1.94)	0.208	
	Allele, C (%)	344 (23.6)	203 (28.2)			
rs699947(2578 C>A)						
	Co-dominant					
	CC	468 (64.3)	179 (49.7)	1.00 (ref)		0.126
	CA	223 (30.6)	137 (38.1)	1.33 (1.05–1.71)	0.012	
	AA	37 (5.1)	44 (12.2)	2.35 (1.57–3.16)	< 0.001	
	Dominant					
	CC	468 (64.3)	179 (49.7)	1.00 (ref)		
	CA+AA	260 (35.7)	181 (50.3)	1.70 (1.16–2.31)	< 0.001	
	Allele, A (%)	297 (20.4)	225 (31.3)			
rs833061(460 C>T)						
	Co-dominant					
	CC	417 (57.3)	185 (51.4)	1.00 (ref)		0.138
	CT	258 (35.4)	139 (38.6)	1.22 (0.80–1.78)	0.402	
	TT	53 (7.3)	36 (10.0)	1.45 (0.84–2.10)	0.381	
	Dominant					
	CC	417 (57.3)	185 (51.4)	1.00 (ref)		
	CT+TT	311 (42.7)	175 (48.6)	1.29 (0.82–1.90)	0.357	
	Allele, T (%)	364 (25.0)	211 (29.3)			
rs25648 VEGF-7C/T						
	Co-dominant					
	CC	421 (57.8)	186 (51.7)	1.00 (ref)		0.342
	CT	259 (35.6)	137 (38.0)	1.24 (0.81–1.83)	0.613	
	TT	48 (6.6)	37 (10.3)	1.57 (0.75–2.40)	0.542	
	Dominant					
	CC	421 (57.8)	186 (51.7)	1.00 (ref)		
	CT+TT	307 (42.2)	174 (48.3)	1.26 (0.79–1.99)	0.441	
	Allele, T (%)	355 (24.4)	211 (29.3)			
**rs833052**						
	Co-dominant					
	CC	476 (65.4)	180 (50.0)	1.00 (ref)		0.310
	CA	220 (30.2)	144 (40.0)	1.49 (1.25–1.87)	< 0.001	
	AA	32 (4.4)	36 (10.0)	2.10 (1.41–2.86)	< 0.001	
	Dominant					
	CC	476 (65.4)	180 (50.0)	1.00 (ref)		
	CA+AA	252 (34.6)	180 (50.0)	1.65 (1.23–2.12)	< 0.001	
	Allele, A (%)	284 (19.5)	216 (30.0)			
rs3025039						
	Co-dominant					
	CC	463 (63.6)	202 (56.1)	1.00 (ref)		
	CT	227 (31.2)	126 (35.0)	1.32 (0.92–1.81)	0.086	
	TT	38 (5.2)	32 (8.9)	1.52 (0.83–2.35)	0.107	
	Dominant					
	CC	463 (63.6)	202 (56.1)	1.00 (ref)		
	CT+TT	265 (36.4)	158 (43.9)	1.38 (0.90-1.87)	0.094	
	Allele, T (%)	303 (20.8)	190 (26.4)			

* Adjusted for gender, age, smoking and alcohol status, BMI. Bonferroni correction threshold: *p* < 0.00833.

GMDR model was used to screen the best interaction combination among 6 SNPs within VEGF gene, and find the gene- gene and gene- smoking interaction on bladder cancer risk. Table 3 summarized the results obtained from GMDR analysis and indicated a potential interaction between rs2010963 and smoking on bladder cancer risk (*p* = 0.0107). Overall, the cross-validation consistency of this model was 9/10, and the testing accuracy was 60.11%. But we did not find a significant any-locus model among SNPs. We also conducted interaction analysis for the significant GMDR model by using logistic regression. We found that current smokers with rs2010963- GC or CC genotype within VEGF gene have the highest bladder cancer risk, compared to never smokers with rs2010963- GG genotype, OR (95%CI) = 3.25 (1.71–4.83), after covariates adjustment (Table 4).

**Table 3:** GMDR analysis on the best gene–gene and gene- smoking interaction models

Locus no.	Best combination	Cross-validation consistency	Testing accuracy	*p*-values
Gene- gene interactions*				
2	rs2010963 rs833052	9/10	0.5399	0.0547
3	rs2010963 rs833052 rs833061	8/10	0.5399	0.1719
4	rs2010963 rs833052 rs833061 rs25648	7/10	0.5399	0.3770
5	rs2010963 rs833052 rs833061 rs25648 rs699947	6/10	0.4958	0.4258
6	rs2010963 rs833052 rs833061 rs25648 rs699947 rs3025039	5/10	0.4958	0.6230
Gene- smoking interactions ^*^				
2	rs2010963 Smoking	9/10	0.6011	0.0107
3	rs2010963 rs833052 Smoking	9/10	0.5399	0.1719
4	rs2010963 rs833052 rs833061 Smoking	8/10	0.4958	0.3770
5	rs2010963 rs833052 rs833061 rs25648 Smoking	7/10	0.4958	0.4258
6	rs2010963 rs833052 rs833061 rs25648 rs699947 Smoking	6/10	0.4958	0.6230
7	rs2010963 rs833052 rs833061 rs25648 rs699947 rs3025039 Smoking	5/10	0.4958	0.9893

* Adjusted for gender, age, smoking, drinking and BMI for gene- gene interaction analysis.

**Adjusted for gender, age, drinking and BMI for gene- smoking interaction analysis.

**Table 4:** Analysis for gene- smoking interaction by using logistic regression

rs2010963	Smoking	OR (95% CI)*	*P*-values
GG	Never	1.00	–
GC+CC	Never	1.35 (0.96–1.87)	0.108
GG	Current	1.46 (1.07–2.02)	0.021
GC+CC	Current	3.25 (1.71–4.83)	< 0.001

* Adjusted for gender, age, drinking and BMI.

Bonferroni correction threshold: *p* < 0.0125.

Pairwise LD analysis between SNPs was performed and the D’ values were shown in Table 5. Just the D’ value between rs2010963 and rs833052 was more than 0.8. The most common haplotype was rs2010963- G and rs833052- C haplotype, the frequencies of which were 0.4234 and 0.4915 in case and control group, respectively. Haplotype containing the rs2010963- C and rs833052- A alleles were associated with a statistically increased bladder cancer risk, OR (95%CI) = 2.21 (1.12 – 3.42) (Table 6).

**Table 5:** the D’ values among 6 SNPs within VEGF gene for the linkage disequilibrium test

SNPs	D’ values				
	rs833052	rs833061	rs25648	rs699947	rs3025039
rs2010963	**0.848**	0.423	0.271	0.054	0.0023
rs833052	-	0.312	0.028	0.108	0.402
rs833061	-	-	0.354	0.0025	0.371
rs25648	-	-	-	0.301	0.149
rs699947	-	-	-	-	0.004

**Table 6:** Haplotype analysis on association between VEGF gene and bladder cancer risk

Haplotypes	rs2010963	rs833052	Frequencies		OR(95%CI)	*p*-values*
			Case group	Control group		
H1	**G**	**C**	0.4234	0.4915	1.00	—
H2	**G**	**A**	0.2703	0.2681	1.21 (0.85–1.66)	0.421
H3	**C**	**C**	0.1995	0.1862	1.46 (0.96–1.98)	0.108
H4	**C**	**A**	0.1068	0.0542	2.21 (1.12–3.42)	0.0002

* Adjusted for gender, age, smoking, drinking and BMI; Bonferroni correction threshold: *p* < 0.0125.

## DISCUSSION

In this study, we found that the A allele of rs699947 and the A allele of rs833052 within VEGF gene were significantly associated with increased bladder cancer risk. The VEGF gene was located on chromosome 6p21.3 and consisted of 8 exons exhibiting alternate splicing to form a family of proteins [14]. Some studies have reported that SNPs within VEGF were associated with many types of cancer, such as oral, breast, glioma, colorectal and lung [15–17]. Recently, some studies focused on the association between VEGF gene polymorphisms and bladder cancer risks were reported, but they concluded inconsistent results. VEGF is pivotal to the neovascular growth required to sustain solid tumor progression [18]. Crew et al [19] have demonstrated the role of elevated urinary levels of VEGF on bladder cancer specimens. Urquidi et al [20] suggested that VEGF could be a valuable addition to voided urine sample analysis for the detection of BCa. However, another study [21] conducted in Canary Islands and Spain indicated that subjects with the VEGF genotype might be not significantly associated with risk of bladder cancer. García-Closas et al. [22] indicated that three SNPs in the promoter region were associated with increased risk for bladder cancer, but a polymorphism in intron 2 was associated with reduced risk. A study from Japan [23] suggested that the serum VEGF level correlates significantly with muscular invasiveness, VEGF promotes tumor proliferation and invasion through VEGFR-2. Jaiswal et al [24] indicated that genotypes of VEGF- rs699947 and rs35569394 polymorphism in the promoter region of VEGF gene may affect the disease susceptibility, significant associations of bladder cancer risk with heterozygous CA genotype (1.69-folds) in VEGF- rs699947 and heterozygous genotype of VEGF rs35569394 were observed, but VEGF- rs35569394 genotype showed reduced risk for bladder cancer. Yang et al. [25] conducted a study for Chinese population and indicated that the rs3025039 and rs1570360 gene polymorphisms were not found to be correlated with the risk of bladder cancer or its progression, but the VEGF rs833052 C/A polymorphism may be associated with a modest increase in the risk of bladder cancer. Although the fore- mentioned two studies have obtained the similar results with that in current study, sample size in study by Jaiswal et al was relatively smaller than that in our study. The problem on sample size was not exist in the study by Yang et al, but the SNPs included in this study was less, so some others SNPs which were associated with bladder cancer may be missed. VEGF or VEGF receptors (VEGFR) expression and the exact function of VEGF/VEGFR receptor signaling on bladder cancer development remain unclear. Zhang et al. [26] suggested that VEGF expression levels were significantly associated with tumor stage, tumor grade and lymph node metastasis (all *P* < 0.05), it means that VEGF may be promising candidates for use as diagnostic and prognostic molecular biomarkers for BC. Earlier study showed that there is association between grade/stage and VEGF expression in bladder cancer [27].

Bladder cancer susceptibility was influenced by both genetic and environment factors, and previously several environmental factors associated with bladder cancer were reported, including cigarette smoking, exposure to industrial aromatic amines and the uptake of drugs [28]. Studies [11, 12] have verified that tobacco smoking plays a crucial role in the etiology of bladder cancer. The risk of bladder cancer in cigarette smokers was 2 to 6-folds higher compared to that in non-smokers, as several compounds in cigarettes may cause genotoxic events in the urothelium [29], and several genes have been reported interaction with smoking on bladder cancer susceptibility [30], however, no study focused on impact of interaction between VEGF gene and smoking on bladder cancer risk. In current study, the rate of smoking was higher in bladder cancer cases than that in controls, it means that smoking was positively associated with bladder cancer risk, so in this study we not only investigated gene- gene interaction on bladder cancer risk, but also gene- smoking interaction. We found a potential interaction between rs2010963 and smoking on bladder cancer risk, current smokers with rs2010963- GC or CC genotype within VEGF gene have the highest bladder cancer risk, compared to never smokers with rs2010963- GG genotype. We also checked the LD among these SNPs and found that the D’ value between rs2010963 and rs833052 was relatively larger (more than 0.8). So the haplotype analysis was conducted for the two SNPs, and found that haplotype containing the rs2010963- C and rs833052- A alleles were associated with a statistically increased bladder cancer risk.

There several limitations in our study. Firstly, the sample size for this study was relatively small, although it has met the requirement; Secondly, more SNPs within VEGF gene should been included in the future analysis. Thirdly, all controls were selected from a community-based chronic non-communicable diseases screening program, therefore, a selection bias could not be avoided and the subjects may not be representative of the general population. Finally, we did not evaluate the relationships of these SNPs with the plasma levels of VEGF, which may potentially reflect the disease state of patients.

In conclusion, we found that the A allele of rs699947 and the A allele of rs833052 within VEGF gene, interaction between rs2010963 and smoking, and haplotype containing the rs2010963- C and rs833052- A alleles were all associated with increased bladder cancer risk.

## MATERIALS AND METHODS

### Subjects

**Figure 1:**
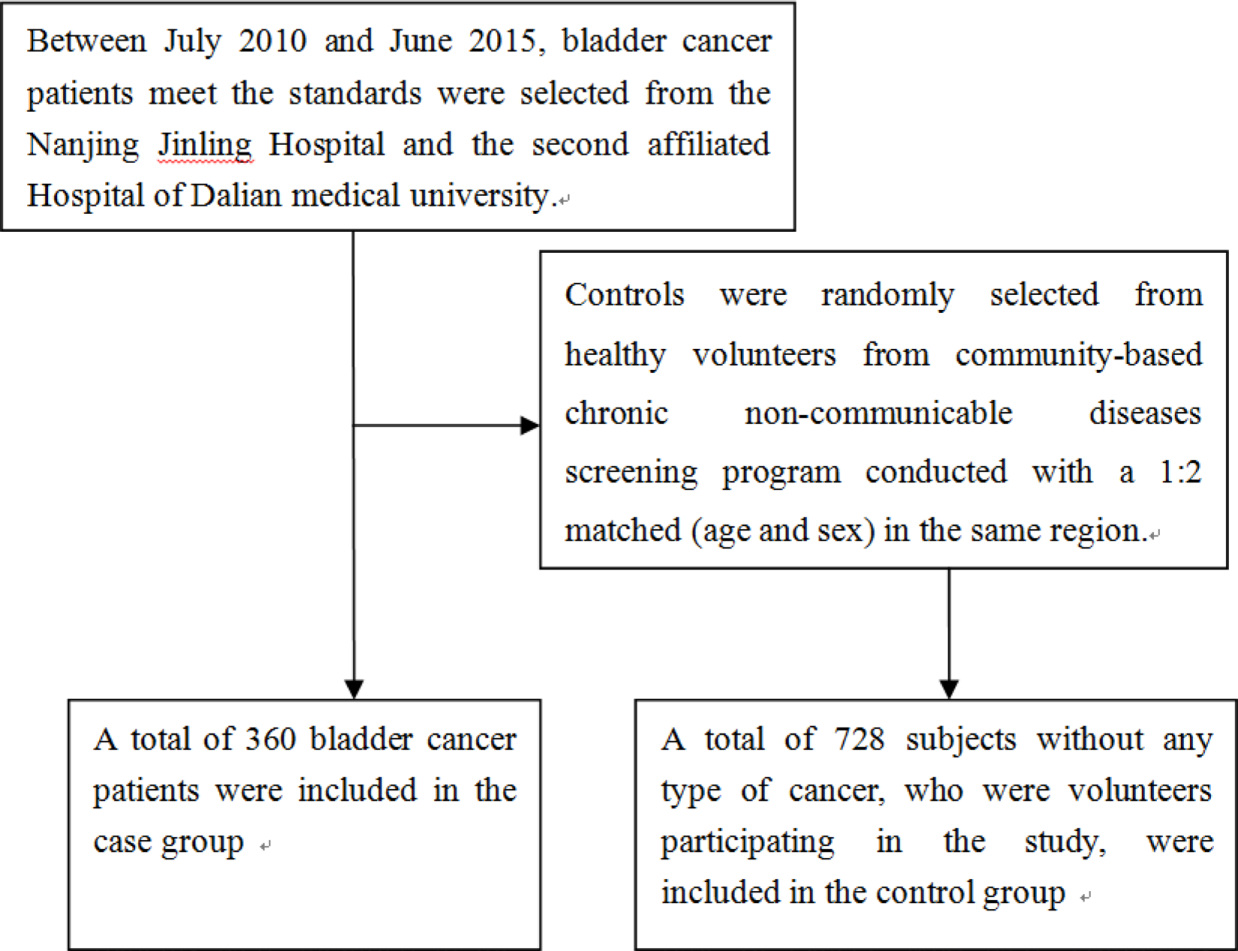
A flowchart on study population selection and exclusion

Between July 2010 and June 2015, bladder cancer patients met the standards were selected from the Nanjing Jinling Hospital and the second affiliated Hospital of Dalian medical university. All the cases were histopathologically confirmed and staged according to the tumor-node-metastasis staging system of the Union for International Cancer Control [31]. The tumors were graded according to the World Health Organization classification. Controls were randomly selected from healthy volunteers from community-based chronic non-communicable diseases screening program, and conducted with a 1:2 matched (age and sex) in the same region. Consequently, a total of 360 diagnosed bladder cancer cases and 728 control participants were included in current study (Figure 1). Information regarding gender, age, tobacco smoking and tumor histopathology was obtained from medical records. Body weight and height were measured. Body mass index (BMI) was calculated as weight in kilograms divided by the square of the height in meters. Current cigarette smokers were those who self-reported smoking cigarettes at least once a day for 1 year or more. Alcohol consumption was expressed as the sum of milliliters of alcohol per week from wine, beer, and spirits. Informed consent was obtained from all participants. This study was approved by the ethics committee of Nanjing University School of Medicine.

### Genomic DNA extraction and genotyping

The SNPs were selected based on the NCBI database (http://www.ncbi.nlm.nih.gov/projects/SNP) according to the following three criteria: 1) located in a gene fragment that could have functional effects; 2) MAF more than 5%; 3) previously reported associations with bladder cancer, but were not well studied. Taking into account the limited human resources and financial resources, just 6 SNPs within VEGF gene were selected for genotyping, including: rs2010963, rs833052, rs833061, rs25648, rs699947 and rs3025039. Genomic DNA from participants was extracted from EDTA-treated whole blood, using the DNA Blood Mini Kit (Qiagen, Hilden, Germany) according to the manufacturer's instructions and stored at −20°C until use. The genotypes of selected 6 SNPs were detected by polymerase chain reaction-restriction fragment length polymorphism (PCR–RFLP) method. The nucleotide sequence of primers and description for the 6 SNPs within VEGF gene were shown in Table 7. The PCR–RFLP program for the SNPs amplification consisted of an initial denaturation step at 95°C for 2 min, followed by 35 cycles of 30 sec at 95°C, 30 sec at 67°C, 30 sec at 55°C and a final elongation at 72°C for 5 min. Genotyping results were confirmed by randomly assaying 10% of the original specimens for replication to exclude genotyping errors. There were no discrepancies between genotypes determined in duplicate.

**Table 7:** Description and probe sequence for 6 SNPs used for PCR analysis

SNP ID	Chromosome	Functional Consequence	Nucleotide substitution	Primer sequences	Restriction enzyme
**rs2010963 +405 G > C**	6:43770613	Upstream variant 2KB, utr variant 5 prime	G > C	F:5′-TTGCTTGCCATTCCCCACTTGA-3′R: 5′- CCGAAGCGAGAACAGCCCAGA-3′	Faq I
rs833061460 C > T	6:43769749	Upstream variant 2KB	C > T	F:5′-TGAGTGTGTGCGTGTGGGGTTGAGCG-3′R: 5′- AGAGCCGTTCCCTCTTTGCTAG-3′	Hinp I
rs6999472578 C > A	6:43768652	Upstream variant 2KB	C > A	F:5′- GGCCTTAGGACACCATACC-3′R: 5′-CACAGCTTCTCCCCTATCC- 3′	Bgl II
rs25648VEGF-7C/T	6:43771240	synonymous codon, upstream variant 2KB, utr variant 5 prime	C > T	Forward: 5′- GCACTGACTCTGGCTCTGAC-3′Reverse: 5′- ACCCTCCTGCTCCTGTGGCT-3′	Mnl I
rs833052	6:43755598	-	C > A	Forward:5′-ACGTTGGATGTAGAAAACACA GCGACTGGC-3′Reverse: 5′-ACGTTGGATGTACCAGGCTTG AAATGACAG-3′	*Acl* I
rs3025039	6:43784799	Utr variant 3 prime	C > T	Forward: 5′-GCCCGAGCCGCGTGTGGAA-3′ Reverse: 5′-GCCCGAGCCGCGTGTGGAG-3′	*Nla* III

### Statistical analysis

SPSS 22.0 software package (SPSS Inc, Chicago) for Windows 7 was used for all statistical analysis in this study. The means and standard deviations (SD) were calculated for normally distributed continuous variables and compared by using Student's t test. Percentages were calculated for categorical variables and were analyzed using χ^2^ test. Hardy-Weinberg equilibrium and allele frequencies in cases and controls were calculated using SNPstats (http://bioinfo.iconcologia.net/SNPstats). Logistic regression was performed to investigate association between 6 SNPs within VEGF gene, additional gene- smoking interaction on bladder cancer risk. All reported *p*-values were two-tailed, and those less than 0.05 were considered statistically significant.

Generalized multifactor dimensionality reduction (GMDR) [32] was used to screen the best interaction combination among SNPs and smoking. The cross-validation consistency score is a measure of the degree of consistency with which the selected interaction is identified as the best model among all possibilities considered. The testing balanced accuracy is a measure of the degree to which the interaction accurately predicts case–control status with scores between 0.50 (indicating that the model predicts no better than chance) and 1.00 (indicating perfect prediction). Finally, a sign test or a permutation test (providing empirical *p*-values) for prediction accuracy can be used to measure the significance of an identified model.

## References

[1] Kakehi Y, Hirao Y, Kim WJ, Ozono S, Masumori N, Miyanaga N, Nasu Y, Yokomizo A (2010). Bladder Cancer Working Group report. Jpn J Clin Oncol.

[2] Yee DS, Ishill NM, Lowrance WT, Herr HW, Elkin EB (2011). Ethnic differences in bladder cancer survival. Urology.

[3] Parkin DM (2004). International variation. Oncogene.

[4] Hainaut P, Pfeifer GP (2001). Patterns of p53 G > T transversionsin lung cancers reflect the primary mutagenic signature of DNA-damage by tobacco smoke. Carcinogenesis.

[5] Volanis D, Kadiyska T, Galanis A, Delakas D, Logotheti S, Zoumpourlis V (2010). Environmental factors and genetic susceptibility promote urinary bladder cancer. Toxicol Lett.

[6] Ferrara N (2004). Vascular endothelial growth factors as a target for anticancer therapy. Oncologist.

[7] Watson CJ, Webb NJ, Bottomley MJ, Brenchley PE (2000). Identification of polymorphisms within the vascular endothelial growth factor (VEGF) gene: correlation with variation in VEGF protein production. Cytokine.

[8] Bae SJ, Ahn DH, Hong SP, Kang H, Hwang SG, Oh D, Kim NK (2008). Gender-specific association between polymorphism of vascular endothelial growth factor (VEGF 936C > T) gene and patients with stomach cancer. Yonsei Med J.

[9] Kawai Y, Sakano S, Korenaga Y, Eguchi S, Naito K (2007). Associations of single nucleotide polymorphisms in the vascular endothelial growth factor gene with the characteristics and prognosis of renal cell carcinomas. Eur Urol.

[10] Zhou Y, Li N, Zhuang W, Wu X (2011). Vascular endothelial growth factor (VEGF) gene polymorphisms and gastric cancer risk in a Chinese Han population. Mol Carcinog.

[11] Bjerregaard BK, Raaschou-Nielsen O, Sørensen M, Frederiksen K, Christensen J, Tjønneland A, Overvad K, Chapelon FC, Nagel G, Chang-Claude J, Bergmann MM, Boeing H, Trichopoulos D (2006). Tobacco smoke and bladder cancer- in the European Prospective Investigation into Cancer and Nutrition. Int J Cancer.

[12] Freedman ND, Silverman DT, Hollenbeck AR, Schatzkin A, Abnet CC (2011). Association between smoking and risk of bladder cancer among men and women. JAMA.

[13] Liu Y, Wang H, Lin T, Wei Q, Zhi Y, Yuan F, Song B, Yang J, Chen Z (2012). Interactions between cigarette smoking and XPC-PAT genetic polymorphism enhance bladder cancer risk. Oncol Rep.

[14] Vincenti V, Cassano C, Rocchi M, Persico G (1996). Assignment of the vascular endothelial growth factor gene to human chromosome 6p21.3. Circulation.

[15] Zhai R, Gong MN, Zhou W, Thompson TB, Kraft P, Su L, Christiani DC (2007). Genotypes and haplotypes of the VEGF gene are associated with higher mortality and lower VEGF plasma levels in patients with ARDS. Thorax.

[16] Lambrechts D, Storkebaum E, Morimoto M, Del-Favero J, Desmet F, Marklund SL, Wyns S, Thijs V, Andersson J, van Marion I, Al-Chalabi A, Bornes S, Musson R (2003). VEGF is a modifier of amyotrophic lateral sclerosis in mice and humans and protects motoneurons against ischemic death. Nat Genet.

[17] Jin Q, Hemminki K, Enquist K, Lenner P, Grzybowska E, Klaes R, Henriksson R, Chen B, Pamula J, Pekala W (2005). Vascular endothelial growth factor polymorphisms in relation to breast cancer development and prognosis. Clin Cancer Res.

[18] Ferrara N (2002). VEGF and the quest for tumour angiogenesis factors. Nat Rev Cancer.

[19] Crew JP, O'Brien T, Bicknell R, Fuggle S, Cranston D, Harris AL (1999). Urinary vascular endothelial growth factor and its correlation with bladder cancer recurrence rates. J Urol.

[20] Urquidi V, Goodison S, Kim J, Chang M, Dai Y, Rosser CJ (2012). Vascular Endothelial Growth Factor, Carbonic Anhydrase 9, and Angiogenin as Urinary Biomarkers for Bladder Cancer Detection. Urology.

[21] Henríquez-Hernández LA, Navarro P, Luzardo OP, Alvarez-León EE, Boada LD, Zumbado M, Pestano J, Suárez JR, Chesa N, Almeida M, Valerón PF (2012). Polymorphisms of glutathione S-transferase and, MDR1 and VEGF genes as risk factors of bladder cancer: A case-control study. Urol Oncol.

[22] García-Closas M, Malats N, Real FX, Yeager M, Welch R, Silverman D, Kogevinas M, Dosemeci M, Figueroa J, Chatterjee N, Tardón A, Serra C, Carrato A (2007). Large-Scale Evaluation of Candidate Genes Identifies Associations between VEGF Polymorphisms and Bladder Cancer Risk. PLoS Genet.

[23] Nakanishi R, Oka N, Nakatsuji H, Koizumi T, Sakaki M, Takahashi M, Fukumori T, Kanayama HO (2009). Effect of Vascular Endothelial Growth Factor and Its Receptor Inhibitor on Proliferation and Invasion in Bladder Cancer. Urol Int.

[24] Jaiswal PK, Tripathi N, Shukla A, Mittal RD (2013). Association of single nucleotide polymorphisms in vascular endothelial growth factor gene with bladder cancer risk. Med Oncol.

[25] Yang Y, Zhang X, Song D, Wei J (2014). Association between vascular endothelial growth factor gene polymorphisms and bladder cancer risk. Mol Clin Oncol.

[26] Zhang HH, Qi F, Cao YH, Zu XB, Chen MF (2015). Expression and clinical significance of microRNA-21, maspin and vascular endothelial growth factor-C in bladder cancer. Oncol Lett.

[27] Wang S, Xia T, Zhang Z, Kong X, Zeng L, Mi P, Xue Z (2000). Expression of VEGF and tumor angiogenesis in bladder. Zhonghua Wai Ke Za Zhi.

[28] Cohen SM, Shirai T, Steineck G (2000). Epidemiology and etiology of premalignant and malignant urothelial changes. Scand J Urol Nephrol Suppl.

[29] Brennan P, Bogillot O, Cordier S, Greiser E, Schill W, Vineis P, Lopez-Abente G, Tzonou A, Chang-Claude J, Bolm-Audorff U, Jöckel KH, Donato F, Serra C (2000). Cigarette smoking and bladder cancer in men: a pooled analysis of 11 case-control studies. Int J Cancer.

[30] Garcia-Closas M, Rothman N, Figueroa JD, Prokunina-Olsson L, Han SS, Baris D, Jacobs EJ, Malats N, De Vivo I (2013). Common Genetic Polymorphisms Modify the Effect of Smoking on Absolute Risk of Bladder Cancer. Cancer Res.

[31] Deng J, Zhang R, Pan Y, Wang B, Wu L, Jiao X, Bao T, Hao X, Liang H (2014). Comparison of the staging of regional lymph nodes using the sixth and seventh editions of the tumor-node-metastasis (TNM) classification system for the evaluation of overall survival in gastric cancer patients: findings of a case-control analysis involving a single institution in China. Surgery.

[32] Lou XY, Chen GB, Yan L, Ma JZ, Zhu J, Elston RC, Li MD (2007). A generalized combinatorial approach for detecting gene-by gene and gene-by-environment interactions with application to nicotine dependence. Am J Hum Genet.

